# Exploring the application of sildenafil for high-fat diet-induced erectile dysfunction based on interleukin-18-mediated NLRP3/Caspase-1 signaling pathway

**DOI:** 10.1093/sexmed/qfad044

**Published:** 2023-08-25

**Authors:** Bingbing Zhu, Yangjiu Niu, Lipan Niu, Xijia Zhang, Fengxia Liu

**Affiliations:** Department of Human Anatomy, School of Basic Medical Science, Xinjiang Medical University, Urumqi, Xinjiang Uygur Autonomous Region, 830011, China; Department of Human Anatomy, School of Basic Medical Science, Xinjiang Medical University, Urumqi, Xinjiang Uygur Autonomous Region, 830011, China; Department of Human Anatomy, School of Basic Medical Science, Xinjiang Medical University, Urumqi, Xinjiang Uygur Autonomous Region, 830011, China; Department of Human Anatomy, School of Basic Medical Science, Xinjiang Medical University, Urumqi, Xinjiang Uygur Autonomous Region, 830011, China; Department of Human Anatomy, School of Basic Medical Science, Xinjiang Medical University, Urumqi, Xinjiang Uygur Autonomous Region, 830011, China

**Keywords:** erectile dysfunction, IL-18, sildenafil, PDE5 inhibitors, NLRP3

## Abstract

**Background:**

Inflammation is a key risk factor for heart disease and has also been linked to erectile dysfunction (ED). Sildenafil is a phosphodiesterase type 5 inhibitor with a strong antioxidant effect. Interleukin (IL)-18 is a proinflammatory factor. Excessive production and release of IL-18 disrupt the balance between IL-18 and IL-18 binding proteins in certain inflammatory diseases, leading to the occurrence of pathological inflammation.

**Aim:**

We evaluated the effects of sildenafil on erectile function in a rat model of high-fat diet–induced ED.

**Methods:**

Male Sprague Dawley rats (6 weeks old) were divided into 5 groups: control, ED, sildenafil, IL-18, and IL-18 + sildenafil. Subsequently, intracavernous pressure and mean arterial pressure were used to assess the erectile function of these rats. The expression of endothelial nitric oxide synthase, pyroptosis factors, and the ratio of smooth muscle cells and collagen fibers were evaluated in the serum and corpora tissue.

**Outcomes:**

Exploring the role and mechanism of sildenafil in ED through NLRP3-mediated pyroptosis pathway.

**Results:**

In comparison to the ED and IL-18 groups, there were statistically significant increases in the ratio of intracavernous pressure to mean arterial pressure, endothelial nitric oxide synthase expression, and the ratio of smooth muscle cells to collagen fibers following sildenafil intervention (*P* < .05). The sildenafil group and IL-18 + sildenafil group also showed statistically significant decreases the expression of NLRP3, caspase-1, and gasdermin D (*P* < .05).

**Clinical Implications:**

Sildenafil can improve erectile dysfunction by inhibiting inflammation.

**Strengths and Limitations:**

Strengths are that the relationship between pyroptosis and ED has been verified through in vitro and in vivo experiments. The limitation is that the conclusions drawn from animal and cells experiments need to be confirmed in clinical research.

**Conclusion:**

Sildenafil may reduce the effect of IL-18–induced inflammation in high-fat diet–induced ED rats through NLRP3/caspase-1 pyroptosis pathway.

## Introduction

Erectile dysfunction (ED), defined as the inability to acquire or maintain a strong enough erection for pleasant sexual engagement, is one of the most common sexual dysfunctions in men.^1^ By 2025, 322 million men worldwide are projected to experience ED in varied degrees.^2^ If ED patients do not receive treatment in time, it not only will reduce patients’ sexual life satisfaction and have an adverse effect on family stability, but may also delay the detection and treatment of various disorders, such as cardiovascular disease.^3^ According to studies, the pathophysiological mechanism of ED is complicated and affected by several factors, including inflammatory response, oxidative stress, vascular alterations, and changes in vasoactive substances.^4^ The primary method of treatment for ED at the moment is phosphodiesterase type 5 inhibitor medication therapy, such as sildenafil (Sil).^5^ According to clinical research, Sil plays a significant part in the treatment of various types of ED and helps improve erectile function in ED patients whether given in regular small doses or high doses when needed.^6^ This medication assists patients to have satisfying sexual encounters by encouraging the creation of endothelial nitric oxide synthase (eNOS), which raises the level of nitric oxide and causes vasodilation in the penis and promotes penile engorgement and erection.^7^

Pyroptosis is a recently identified unique type of procedural proinflammatory cell death.^8^^,^^9^ Following abnormal stimulation, aspartate-specific protease-1 precursor, NLRP3, and apoptosis-associated speck-like protein work together to activate caspase-1. Activated caspase-1 promotes gasdermin D (GSDMD) to cause cell membrane perforation. These gaps in the cell membrane allow inflammatory substances like IL-18 to escape and act on other cells in the body to cause a proinflammatory reaction that results in cell death.^10^^,^^11^ Interleukin 18 (IL-18) originating from macrophages promotes inflammatory action through the nuclear factor kB pathway, and induce interferon γ production in combination with IL-12 on CD4, CD8 T cells, and natural killer cells. In addition, a large number of studies have proved that the pathological studies, clinical diagnosis, and treatment of many diseases (such as autoimmune diseases, inflammatory diseases, and tumors) are closely related to IL-18.^12–14^ It has been demonstrated that orchiectomy’s hypoandrogenic state encourages pyroptosis in endothelial cells of the rat penile corpus cavernosum, impairing erectile function.^15^ Additionally, it was shown that diabetic ED rats’ penile cavernous tissue had higher levels of pyroptosis-related factor expression, and that treating rats with adipose stem cells that had NLRP3 knocked down was an efficient way to restore their erectile function.^16^

As a distinct component of the peripheral vascular system, the endothelial cell layer plays a crucial function in controlling vascular contraction, which is crucial in the development of ED.^17^ The inflammatory response, through the expression of inflammatory factors, can both increase the production of vasoconstrictive molecules such as cyclooxygenase 2 and reduce the synthesis and release of eNOS, resulting in endothelial dysfunction.^18^ According to studies, the development of ED is correlated with the expression of the inflammatory factor IL-18.^19^ A high level of IL-18 expression has also been demonstrated to increase the expression of vascular cell adhesion factors while decreasing the expression of IL-18 binding protein.^20^

The objective of the present study was to investigate the effects of Sil on ED in a rat model of high-fat diet (HFD)–induced ED. In addition, we measured pyroptosis factors to examine if ED can be improved Sil by inhibiting the expression of IL-18.

## Methods

### In Vitro Studies

#### Experimental Animals

The Xinjiang Medical University’s Animal Care and Use Committee gave its approval to all operations (Urumqi, Xinjiang, China). A total of 55 specific pathogen–free (nonspecific pathogen animals) male Sprague Dawley rats and 20 female Sprague Dawley rats at sexual maturity were purchased from the Animal Experiment Center of Xinjiang Medical University, weighing 250 ± 10 g. They were fed in an environment of 22 °C to 24 °C and 45% to 50% humidity for 12 hours with alternating light and dark. For 12 weeks, the HFD group consumed a 45% HFD made up of 20% protein, 35% carbohydrate, and 45% fat from HFK Bioscience in Beijing, China. The rats in the control group only had access to a basic meal. Rats’ body weight was recorded once per month. The HFD male rats were injected with apomorphine and observed for 30 minutes to record the number and latency of erections. The model rate of ED rats was 77%, and these ED rats were randomly divided into the ED group (n = 6), Sil group (n = 6), IL-18 group (n = 6), and IL-18 + Sil group (n = 6). In the Sil group, the ED rats received Sil (20 mg/kg/d; Solarbio). In IL-18 group, recombinant IL-18 protein (200 ng/kg/d; R&D Systems) was intraperitoneally injected into the ED rats. As for IL-18 + Sil group, Sil and recombinant IL-18 protein were administered concurrently. The control group (n = 10) was injected with an equal amount of physiological saline.

#### Apomorphine Experiment

Male rats were placed in a metabolic cage, acclimated to the environment for 10 minutes, kept quiet indoors, and treated with apomorphine that was subcutaneously injected into the skin of the neck, and each rat was observed for 30 minutes after injection. The presence and frequency of erections in the penis were recorded. The presence of congestion of the glans and exposure of the terminal penis was considered as one penile erection, while those without penile erection were considered as HFD-induced ED rats. The percentage of rats with penile erection in each group was the erection rate. 

#### Erectile Function Evaluation

The measurement of mean artery pressure (MAP) and intracavernous pressure (ICP) was used to evaluate erectile function.^21^^,^^22^ Rats’ exposed left carotid arteries were cannulated with a PE-50 tube filled with 250 IU/mL heparinized saline and connected to a pressure channel to continuously measure MAP. A lower midline abdominal incision in rats exposed both the penile crus and cavernous nerve. The left crura of rats were then punctured with a 24-G heparinized (heparin 250 IU/mL) needle attached to a different pressure transducer for measuring physiological changes. The electrostimulation adjacent to the cavernous nerve was conducted utilizing a bipolar electrode and controlled by an electrical stimulator, which was capable of generating monophasic rectangular pulses (the following parameters: voltage 7.5 V, frequency 15 Hz, amplitude 1 ms, and duration 1 min). The stimulation was carried out 3 times every 10 minutes, and continuous simultaneous MAP and ICP recordings were made. The erectile responses were shown using the computed ICP/MAP ratio.

### In Vivo Studies

#### Isolation and identification of human umbilical vein endothelial cells

**Human umbilical vein endothelial cells** (HUVECs) were bought from Procell and then cultured in endothelial cell medium supplemented with fetal bovine serum at 5%, endothelial cell growth supplement at 1%, 100 U/mL penicillin, and 100 mg/mL streptomycin at 37 °C in a humidified atmosphere of 5% CO_2_. After 3 passages, cell identification was carried out with von Willebrand factor (vWF) (Abcam) by immunofluorescence and CD31 by flow cytometry. IL-18 plasmid was transfected into cells using Life-EZ-Trans iLab’s cell transfection reagent (Shanghai Life-iLab Biotech Co) in accordance with the manufacturer’s instructions.

#### MTT assay

The ideal concentration and timing of Sil intervention were determined using the MTT test. In 96-well plates, 1 × 10^4^ cells were seeded each well. Then, Sil was administered to the cells in several groups at concentrations of 0 μmol/L, 1 μmol/L, 3 μmol/L, 5 μmol/L, 10 μmol/L, or 15 μmol/L for 24, 48, and 72 hours. Following treatment, 20 μL of MTT dye solution (5 mg/mL in phosphate-buffered saline [PBS] buffer; Sigma) was added to each well and the wells were then incubated at 37 °C for 4 hours. The supernatant was then decanted and 150 μL of DMSO was substituted. The absorbance was determined using a microtiter plate reader at a wavelength of 490 nm following a 15-minute incubation period with gentle shaking at 37 °C.

### Hematoxylin and Eosin Staining

Tissue portions were submerged in hematoxylin for 2 minutes, washed with distilled water for 1 minute, immersed in eosin for 5 seconds, and rinsed again for 1 minute. A series of alcohol concentrations (30 seconds for each concentration) were used to dehydrate sections. Xylene was used to clean the sections and neutral balsam was used to mount them.

### Flow Cytometry

HUVECs with 80% to 90% confluence were digested and centrifuged at 1600 rpm for 5 minutes. The supernatant was discarded and washed twice with PBS solution. The number of cells was adjusted to 1 × 10^5^ cells/mL, the experimental group was FitC-labeled mice with anti-human CD31 monoclonal antibody, and the negative control group was added with the same type of negative control immunoglobulin G1 κ. The cells were incubated at room temperature and kept away from light for 30 minutes. The purity of HUVECs was detected by flow cytometry.

### Immunofluorescence

When the cell confluence reached 80% to 90%, 1 × 10^4^ cells/mL were inoculated in a 35-mm laser confocal culture dish after digestion and centrifugation, and the expression of vWF was identified by immunofluorescence technique on day 3. Samples were fixed with 4% paraformaldehyde at room temperature for 15 minutes, then washed 3 times with PBS for 5 minutes; precooled PBS of 2.5 mL/L Triton X-100 was placed on ice permeable for 10 minutes and closed with 10 g/L bovine serum albumin for 1 hour. Rabbit anti-human vWF polyclonal antibody (1:250) was added and incubated overnight at 4 °C. The next day, the second antibody immunoglobulin G was incubated at room temperature and away from light for 2 hours. DAPI restained nuclei at room temperature for 5 minutes. A laser confocal microscope was used to take pictures.

### Immunohistological Staining

The center portions of the rat penile shafts were severed and fixed in 4% paraformaldehyde. The samples were embedded and cut into 4-μm sections before mounting on glass slides. The penile tissue sections were incubated with primary antibodies to IL-18 (Affinity Bioscience; 1:800), NLRP3 (Affinity Bioscience; 1:1000), caspase-1 (Proteintech; 1:1000), GSDMD (Proteintech; 1:1000), and eNOS (Proteintech; 1:1000) overnight at 4 °C, while the control sections were incubated without the primary antibodies. Next, the sections were incubated with the goat anti-rabbit secondary antibodies at a 1:200 dilution for 2 hours at room temperature. Quantitative image analysis used computational densitometry using the ImageJ application (National Institutes of Health) and a microscope. The levels of marker expression in each were then normalized as a ratio to control. Ten sections of rat penile tissue were counted, and 6 fields were computed from each section.

### Masson’s Trichrome Staining

According to the Masson staining kit’s instructions (Solarbio), the produced paraffin sections were stained. The findings were assessed using ImageJ software vesion 1.6.0.20, in which blue represented collagen fibers and red represented muscle fibers, and the ratio of blue to the red area was computed.

### Enzyme-Linked Immunosorbent Assay

The supernatants were collected and processed for further testing. The total cholesterol (TC), low-density lipoprotein cholesterol (LDL-C), triglycerides (TG), high density lipoprotein cholesterol (HDL-C), and IL-18 content in penile tissue were determined using an enzyme-linked immunosorbent assay (ELISA) kit that was used according to manufacturer instructions. A microplate reader was used to detect the optical density value of each well sample at 490 nm absorbance.

### Real-Time Quantitative Reverse Transcriptase

RNA was isolated from tissue and cells, reverse transcribed to complementary DNA, and polymerase chain reaction was used to generate IL-18, NLRP3, caspase-1, GSDMD, and eNOS using specified primers. The following ingredients were used in the reaction: 40 cycles of predenaturation at 95 °C for 15 minutes, denaturation at 95°C for 10 seconds, and annealing at 60 °C for 34 seconds. 2^-△△Ct^ Methods were used to determine the relative expression. The IL-18, NLRP3, caspase-1, GSDMD, and eNOS relative expression levels were estimated.

### Western Blotting

Proteins were extracted from the penis and concentrations were analyzed by the BCA Protein Assay kit (Solarbio). Then, equal amounts of proteins were loaded on a sodium dodecyl sulfate polyacrylamide gel for electrophoresis. After transferring the proteins to nitrocellulose membranes, primary antibodies were incubated overnight at 4 °C. GAPDH was used as the loading control. The primary antibodies included IL-18 (1:10000), NLRP3 (1:2000), caspase-1 (1:2000), GSDMD (1:3000), eNOS (1:3000), collagen I (1:2000), and GAPDH (1:6000). The horseradish peroxidase–labeled secondary antibody was diluted in a suitable ratio based on the manufacturer’s instructions. The target protein band and the internal reference band’s gray values were detected using ImageJ software, and the ratio was determined for analysis.

### Statistical Analysis

All experiments were repeated at least 3 times, and the whole data were described as mean ± SD. For analytic method, 1-way analysis of variance test was used. To simulate nonparametric test, a Kruskal-Wallis test was conducted. Differences showing statistical significance were taken as *P* < .05. Data were analyzed using SPSS version 22.0 (IBM Corporation).

## Results

### Changes in biological characterization during rat modeling

#### Body weight and serum lipid profiles

**Figure 1:**
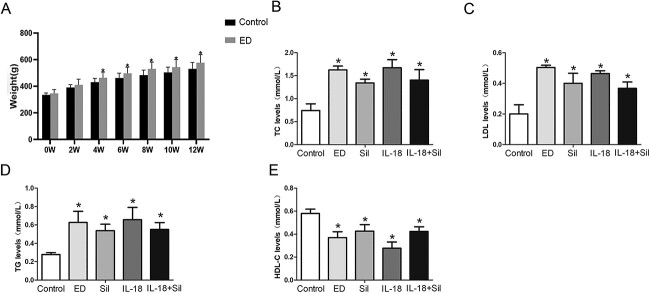
Body weight gain and lipid profiles in each group during the modeling period. (**A**) Changes in body weights of control and erectile dysfunction (ED) rats. The ED rats showed a significant increase in their weight after the induction of the high-fat diet. Histograms showing the mean values of total cholesterol (**B**), low-density lipoprotein cholesterol (**C**), triglycerides (**D**), and high-density lipoprotein cholesterol (**E**) in all groups 12 weeks after the induction of the high-fat diet. Total cholesterol, low-density lipoprotein cholesterol, and triglycerides measured values showed a significant increase, and high-density lipoprotein cholesterol showed a significant decrease in model groups compared with the control group, but there was no significant difference between the model groups. ^*^Significant difference compared with the control group (*P* < .05).

Figure 1 displays the change in body weight as well as blood levels of TC, LDL-C, HDL-C, and TG. Over the duration of the experiment, body weight gain in the HFD-caused ED group was significantly higher than in the control group. Compared with the control group, the levels of TC, LDL-C, and TG were significantly increased and the levels of HDL-C were decreased in the ED group induced by HFD (*P* < .05), but there were no significant differences in the levels of TC, LDL-C, TG, and HDL-C among the 4 model groups. Table 1 shows that after subcutaneous injection of apomorphine, all groups of rats showed increased activity, hair erection, yawning, and other symptoms. The control group showed normal penile erection. The number of erections and erection latency in ED rats were significantly lower than those in the normal group (*P* < .05).

**Table 1:** Change in the number of erections and erection latency of ED rats ($\overline{x}\pm s$).

	Control	ED	Sil	IL-18	IL-18 + Sil
Erectile times	2.00 ± 1.05	0.00 ± 0.00^a^	1.78 ± 2.05^b^	0.00 ± 0.00^a^	2.50 ± 2.07
Erectile latency, s	468.40 ± 455.03	1800.00 ± 0.00^a^	1262.44 ± 657.44^b^	1800.00 ± 0.00^a^	964.5 ± 568.06

Values are mean ± SD.

Abbreviations: ED, erectile dysfunction; IL, interleukin; Sil, sildenafil.

a Compared with the control group, the difference was statistically significant (*P* < .05).

b Compared with the ED group, the difference was statistically significant (*P* < .05).

#### Changes in ICP/MAP ratio in rats in each group after 2 weeks of IL-18 recombinant protein and Sil intervention

**Figure 2:**
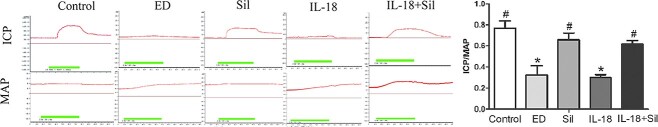
Ratio of rat intracavernous pressure (ICP) to mean arterial pressure (MAP) in each group after 2 weeks of sildenafil (Sil) intervention. (**A**) Representative ICP tracing response to the electronic stimulation of the cavernous nerve (7.5 V, 15 Hz, and 60 seconds’ duration) in erectile dysfunction (ED) rats after injection of Sil. (**B**) The ICP/MAP ratio was calculated for all 5 groups. ^*^Significant difference compared with the control group (*P* < .05). #Significant difference compared with the ED group (*P* < .05).

To evaluate erectile function, the ICP/MAP ratio was utilized. The ICP/MAP ratio was significantly lower in the rats in the ED and IL-18 groups compared with the control group rats, as shown in Figure 2 (*P* < .05). The Sil treatment (Sil and IL-18 + Sil groups) significantly improved the ICP/MAP ratio in ED rats compared with untreated ED rats (*P* < .05), while the Sil treatment group remained with lower ICP/MAP ratio than those in the control group (*P* < .01).

#### Hematoxylin and eosin staining was used to detect the histopathological changes of the penis

**Figure 3:**
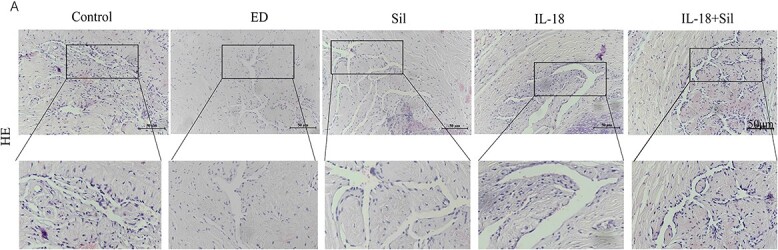
Transverse portions of all rats in all groups’ penises. In the control group, the corpus cavernosum had broad cavernous gaps generated by smooth muscles and collagen fibers. Narrowing of the cavernous spaces in the erectile dysfunction (ED) and interleukin (IL)-18 groups. Results showing almost restoration of cavernous spaces in groups sildenafil (Sil) and IL-18 + Sil, respectively. Hematoxylin and eosin ×200. Scale bar = 50 um.

The control group comprised spongy tissue of rats with broad, irregularly shaped penis cavernous sinus cavity. The surface of the cavernous sinus cavity was covered with many well-arranged and dense endothelial cells, and the endothelial cells on the surface of the cavernous sinus cavity of the ED group were significantly reduced. In the Sil group, endothelial cells on the surface of the cavernous sinus cavity were increased and disordered. In the IL-18 group, endothelial cells on the surface of the cavernous sinus cavity were significantly reduced (*P* < .05). The endothelial cells on the surface of the cavernous sinus cavity increased in the IL-18 + Sil group (*P* < .05). The results indicated that a HFD induced the decrease of endothelial cells on the surface of the cavernous sinus cavity of the penis in ED rats, and Sil could promote the increase of endothelial cells on the surface of the cavernous sinus cavity of the penis in ED rats (Figure 3).

#### Immunohistochemical detection of expression of pyroptosis-related proteins and eNOS in the cavernous tissue of rat penis in various groups

**Figure 4:**
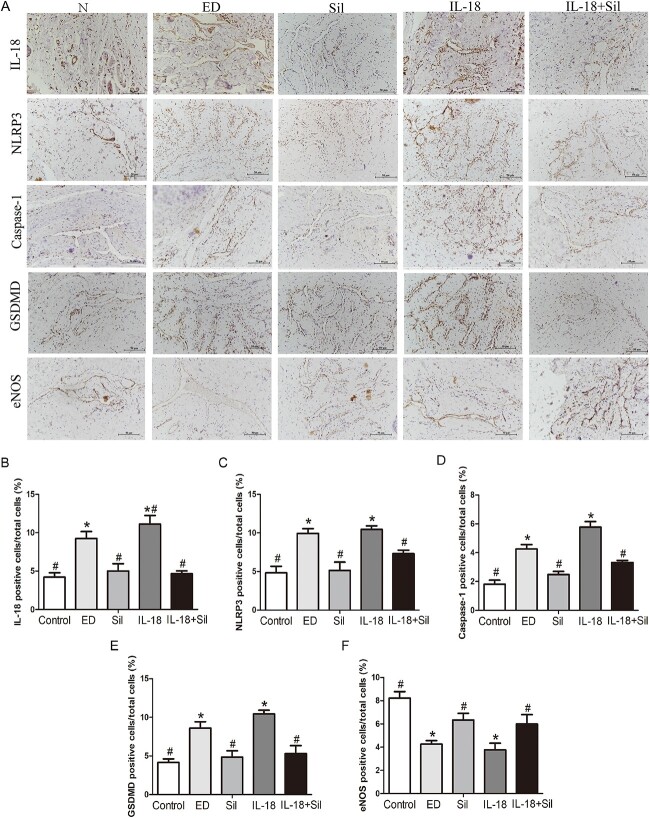
Transverse histological sections of the penises of all rats in all groups were immunohistochemically stained. (**A**) Compared with control group, protein expression of interleukin (IL)-18, NLRP3, caspase-1, and gasdermin D (GSDMD) were increased in the erectile dysfunction (ED) group and IL-18 group, while protein expressions of endothelial nitric oxide synthase (eNOS) were decreased (*P* < .05). (**B**–**F**) Quantitative analysis. ^*^Significant difference compared with the control group (*P* < .05). #Significant difference compared with the ED group (*P* < .05).

As shown in Figure 4, immunohistochemical staining of IL-18, NLRP3, caspase-1, and GSDMD showed increased expression predominately located in the cavernous sinus of the penis of ED rats, which decreased after Sil treatment. However, eNOS-positive cells were diffusely distributed throughout the penis and were usually observed on the periphery of the penis. The expression of eNOS in the cavernous sinus of the ED group and the IL-18 group was significantly reduced (*P* < .05) when compared with the control group. However, the Sil group’s expression of eNOS in the cavernous sinus was significantly higher (*P* < .05) than that of the ED group. The expression of eNOS in the cavernous sinus of the IL-18 + Sil group was likewise significantly higher (*P* < .05) than that of the IL-18 group in comparison. These findings showed that Sil administration could enhance eNOS expression in the penile tissue, hence improving endothelial dysfunction in ED rats.

#### Masson staining to detect each rat corpus cavernosum tissue fibrosis degree

**Figure 5:**
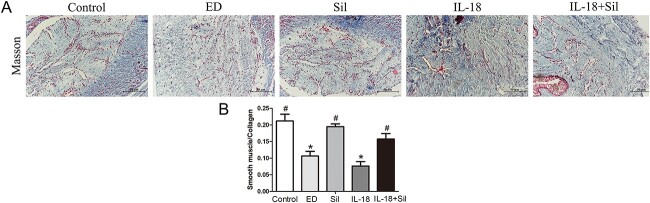
Masson staining to detect the degree of fibrosis in the cavernous tissue of the penis in each group of rats. (**A**) In compared with the control rats, animals in the erectile dysfunction (ED) and interleukin (IL)-18 groups had lower smooth muscle/collagen ratio. Collagen expression in the sildenafil (Sil) and IL-18 + Sil groups is quite similar to that in the control group. (**B**) Quantitative analysis. Masson trichrome stain ×400. Scale bar = 50 um. ^*^Significant difference compared with the control group (*P* < .05). ^#^Significant difference compared with the ED group (*P* < .05).

The smooth muscle and collagen contents in the corpus cavernosum were observed by Masson’s trichrome staining. As shown in Figure 5, the smooth muscle contents were higher in the Sil group than in the ED group (*P* < .05). The smooth muscle contents were higher in the IL-18 + Sil group than in the IL-18 group (*P* < .05). After the Sil injection, α-smooth muscle actin expression level was elevated in the corpus cavernosum, indicating that smooth muscle and angiogenesis increased in injured tissues. These results indicated that Sil had a more positive influence on inflammation, and vascular repair in the ED rats improved.

#### Expression of pyroptosis-related proteins and eNOS in the cavernous tissue of rat penis by real-time quantitative polymerase chain reaction

**Figure 6:**
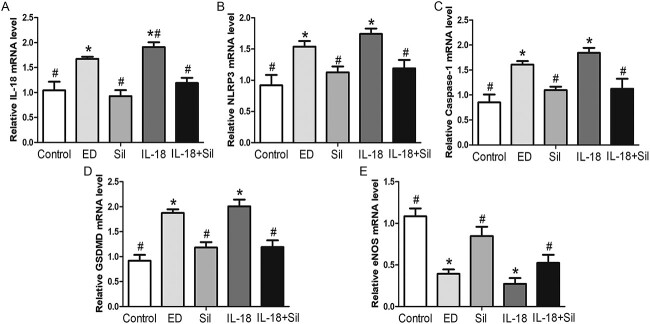
Real-time quantitative polymerase chain reaction detection of pyroptosis-related factors and endothelial nitric oxide synthase (eNOS) messenger RNA (mRNA) levels in the penile tissue of rats in each group. (**A**–**E**) Real-time quantitative polymerase chain reaction of the mRNA levels interleukin (IL)-18, NLRP3, caspase-1, gasdermin D (GSDMD), and eNOS in penile tissue of each group. The erectile dysfunction (ED) and IL-18 groups rats showed increased IL-18, NLRP3, caspase-1, and GSDMD mRNA levels and decreased eNOS mRNA levels in comparison with the control rats. ^*^Significant difference compared with the control group (*P* < .05). ^#^Significant difference compared with the ED group (*P* < .05).

As seen in Figures 6A to 6D, in a quantitative analysis, IL-18, NLRP3, caspase-1, and GSDMD expression in the cavernous sinus of the IL-18 group was significantly higher than that in the control group (*P* < .05), and compared with the ED group, there was no statistically significant difference in the expression of these proteins in the penis. IL-18, NLRP3, caspase-1, and GSDMD messenger RNA (mRNA) expression in the penile tissue of the IL-18 + Sil group was significantly lower than that of the IL-18 group following Sil treatment (*P* < .05). These findings imply that rats with HFD-induced ED have elevated expression of pyroptosis-related factors in the penile tissue, and that Sil treatment reduces this expression to treat ED. However, as seen in Figure 6E, When compared with the control group, the expression of eNOS mRNA was significantly downregulated in both the ED and IL-18 groups (*P* < .05). The expression of eNOS mRNA in the Sil group was significantly higher than that of the ED group (*P* < .05). Similar to the IL-18 group, the IL-18 + Sil group similarly significantly (*P* < .05) enhanced the expression of eNOS mRNA in the cavernous sinus. These findings imply that Sil therapy may help treat ED by inhibiting the expression of inflammatory factors in rat penile tissue.

#### Expression of pyroptosis-related proteins and eNOS in the cavernous tissue of rat penis by Western blot

**Figure 7:**
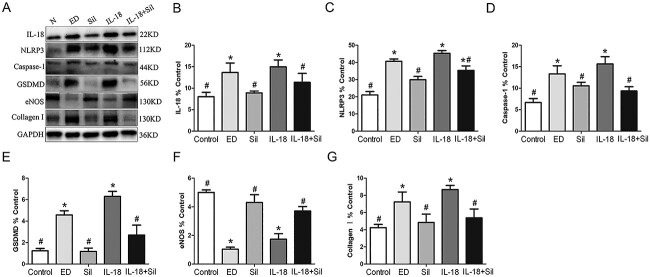
Western blotting detection of pyroptosis-related factors and endothelial nitric oxide synthase (eNOS) protein expressions in the penile tissue of rats in each group. Western blotting (**A**) and quantification (**B**–**G**) of the protein expression of interleukin (IL)-18, NLRP3, caspase-1, gasdermin D (GSDMD), eNOS, and collagen I in penile tissue of each group. The ED and IL-18 groups rats showed increased IL-18, NLRP3, caspase-1, GSDMD, and collagen I protein expression and decreased eNOS protein expression in comparison with the control rats. ^*^Significant difference compared with the control group (*P* < .05). ^#^Significant difference compared with the ED group (*P* < .05).

As shown in Figure 7, compared with the control group, IL-18, NLRP3, caspase-1, GSDMD, and collagen I proteins showed increased expression predominately located in the penis of ED rats, which decreased after Sil treatment, and the difference was statistically significant (*P* < .05). IL-18, NLRP3, caspase-1, GSDMD, and collagen I proteins expression of the IL-18 + Sil group was significantly lower than that of the IL-18 group following Sil treatment (*P* < .05). However, the expression of eNOS in the ED group and the IL-18 group was significantly reduced (*P* < .05) when compared with the control group. The expression of eNOS was significantly higher in the Sil group when compared with the ED group (*P* < .05). Similar to the IL-18 group, the IL-18 + Sil group similarly significantly (*P* < .05) enhanced the expression of eNOS proteins in the cavernous sinus. Sil treatment could improve inflammation, indicating that the activation of pyroptosis in the penis of ED was significantly inhibited by Sil.

### The endothelial injured model

#### Isolated HUVEC morphology and phenotypic characterization

The majority of primary isolated HUVECs were clustered together, and a few cells were scattered. The morphology of scattered cells was typical of cobblestone. Many of the cells in the cluster were long spindles that crawled out from the middle. Each cell cluster was chrysanthemum shaped. After the third generation, the connections between the cells increased, and some cells became larger after the 10th generation (Supplementary Figure 8A). Flow cytometry showed that 98.3% of the cells were positive for CD31 surface antigen, which further indicated that HUVECs with high purity were obtained by this method (Supplementary Figure 8B). Immunofluorescence of vWF showed that 95% of the cells were fluorescent positive. Red fluorescent particles were evenly distributed in the cytoplasm of the cells, and the nuclei were stained blue by DAPI. The fluorescence of vWF-positive cells was mainly concentrated on the cell membrane and intercellular junctions, which is consistent with the cell membrane protein identity of vWF (Supplementary Figure 8C).

#### MTT assay to detect the effect of different concentrations of Sil intervention on the growth of HUVECs

MTT results showed that there was no significant difference among the groups at 24 hours. Compared with the control group, 48-hour treatment with 15 μmol/L Sil significantly inhibited the growth of HUVECs, and the difference was statistically significant (*P* < .05). The 72-hour treatment with 10 μmol/L and 15 μmol/L Sil significantly inhibited the growth of HUVECs, and the difference was statistically significant (*P* < .05). Cell activity results showed that the fatality rate reached 53% at 48 hours after 10 μmol/L Sil intervention. According to our experimental results, we believe that 10 μmol/L Sil intervention for 48 hours is the best intervention plan (Supplementary Figure 9).

#### ELISA to detect the expression of IL-18 between groups of HUVECs

According to the ELISA experiment results, the IL-18 group’s IL-18 expression was much higher than that of the control group, and this difference was statistically significant (*P* < .05). However, there was no discernible difference in the expression of IL-18 between the Sil group, the control group, and the blank group. By transfecting an IL-18 overexpression plasmid, Sil could reduce the rise in IL-18 expression in HUVECs (Supplementary Figure 10).

#### Expression of pyroptosis-related proteins and eNOS in each group of HUVECS by real-time quantitative polymerase chain reaction

As shown in Supplementary Figure 11, when compared with the control group, in the IL-18 group, the mRNA levels of IL-18, caspase-1, NLRP3, and GSDMD were much higher, and these differences were statistically significant (*P* < .05). The mRNA levels of IL-18, caspase-1, NLRP3, and GSDMD in the Sil intervention groups (Sil and IL-18 + Sil groups) were significantly lower than that of the ED and IL-18 groups, with statistically significant differences (*P* < .05). The results of this study indicate that Sil can decrease the expression of IL-18, NLRP3, caspase-1, and GSDMD and increase the expression of eNOS to inhibit inflammatory reaction and improve endothelial dysfunction.

#### Expression of pyroptosis-related proteins and eNOS in each group of HUVECS by Western blot

As shown in Supplementary Figure 12, IL-18, NLRP3, caspase-1, and GSDMD expression increased in the IL-18 group as compared with the control group, and the difference was statistically significant (*P* < .05). These protein expressions in the Sil group were lower than those of the IL-18 group, and this difference was statistically significant (*P* < .05), showing that Sil may limit the expression of proteins linked to pyroptosis in cells. The expression of eNOS in the IL-18 group was decreased when compared with the control group, and the difference was statistically significant (*P* < .05). The eNOS protein expression in the Sil group was higher than that of the IL-18 group, and the difference was statistically significant (*P* < .05), suggesting that Sil may play a protective role in vascular endothelium by inhibiting the expression of inflammatory factors.

## Discussion

With the improvement of living standards, the proportion of high-calorie and high-fat food in people’s daily diet has increased, resulting in a rapid increase in the prevalence of dyslipidemia in Chinese adults.^23^ The modeling method of HFD is similar to the formation principle of dyslipidemia caused by human diet, and the modeling method is convenient, which is the common method of fat modeling animals at present.^24^^,^^25^ This study found that elevated serum levels of TC, TG, and LDL-C can lead to endothelial dysfunction, which is closely related to ED. The ICP/MAP data showed that there was a statistically significant difference between the ICP/MAP ratios of the ED and IL-18 groups and the control group. The ICP/MAP ratio was significantly increased after Sil drug intervention compared with the ED and IL-18 groups. The result showed that Sil treatment may enhance rats’ erectile function. Clinical studies and other ED animal models have shown that Sil was used as a first-line treatment drug for ED and had a good therapeutic impact.^26^^,^^27^

As an important proinflammatory factor, IL-18 can be transcribed and expressed by a variety of cells in organs and tissues (such as endothelial cells, monocytes, etc.), and play multiple biological functions in the process of anti-infection, immune regulation, chronic inflammation, and other diseases.^14^^,^^28^ IL-18 as an important proinflammatory factor can induce the expression of tumor necrosis factor α, IL-1β, and other early inflammatory factors.^29^ IL-18 can promote penile tissue damage and dysfunction by affecting the infiltration of downstream monocytes.^30^ Studies have shown that IL-18 levels increase in ED patients, thus promoting the activation of downstream inflammatory signaling pathways and ultimately promoting the inflammatory destruction of penile tissue.^19^^,^^31^ Second, IL-18 can further induce the secretion of downstream chemokines through the inflammatory cascade reaction, and promote their chemokines to the site of infection, further exacerbating the inflammatory response.^12^^,^^32^ However, The specific mechanism of IL-18–mediated ED is not fully understood.

As a proinflammatory cell death mode, pyroptosis is closely related to the inflammatory response, which is characterized by the assembly and activation of inflammasome, promoting the activation of inflammatory factors and inducing the key protein GSDMD to expose N-terminal domain, thereby destroying the integrity of the cell membrane.^33^^,^^34^ This results in the release of a large number of inflammatory factors into the extracellular and causes cell swelling and rupture.^11^ The most fundamental components of the penis cavernous sinus is the corpus cavernosum, which is made of endothelial cells and covers the surface of the cavity.^35^ The synthesis of eNOS can cause the nitric oxide to diffuse into the surrounding endothelial cells, which increases blood flow to the penis cavernous sinus cavity and causes penis erection.^36^ Increased nitric oxide in the penile tissue facilitates soluble guanylate cyclase acid’s stimulation of guanosine triphosphate into cyclic guanosine monophosphate and activated protein kinase G’s inhibition of calcium ion channel activity on the cell membrane surface, which relaxes cavernous smooth muscle and aids in erection.^37^^,^^38^ Numerous studies have found a clear link between the onset and progression of ED and cell death caused by a variety of factors, including inflammation and oxidative stress.^39^ Currently, according to several research, pyroptosis has a substantial role in the onset and development of erectile dysfunction.^16^^,^^40^ Immunohistochemistry findings in this study revealed that the ED group and the IL-18 group had significantly increased the IL-18, NLRP3, caspase-1, and GSDMD positive area and dramatically decreased the eNOS positive area in the penis compared with the control group. Transfection of siSPTA1 into corpus cavernosum smooth muscle cells resulted in the significant downregulation of mRNA and protein expression of eNOS and significant upregulation of Yes-associated protein, caspase-1, GSDMD, GSDMD-N, IL-18, and IL-1β protein expression levels. The downregulation of SPTA1 in Sprague Dawley rats fed with HFD may induce cell pyroptosis and lead to the decrease of erectile function by activating the Hippo pathway.^41^ According to certain research, stimulating the corpus cavernosum with an NLRP3 activator can increase the production of caspase-1 and IL-18 in mouse penile tissue.^42^ Chen et al^15^ discovered that after orchiectomy, the cavernous sinus endothelial cells of the penis of ED rats showed signs of pyroptosis. In this work, the expression of pyroptosis-related factors at the gene and protein levels in the penile tissue of rats in each group was determined using **real-time quantitative polymerase chain reaction** and Western blot. According to the current research, eNOS expression decreased while IL-18, NLRP3, caspase-1, and GSDMD expression were all higher in the ED and IL-18 groups compared with the control group. However, after Sil intervention, the expression of IL-18, NLRP3, caspase-1, and GSDMD decreased and the expression of eNOS increased. These results suggest that Sil improves erectile dysfunction by inhibiting the expression of inflammatory cytokines and pyroptosis-related factors.

Numerous studies have demonstrated that Sil can significantly lower IL-18 levels of burned rat model heart tissue and that Sil treatment can lower NLRP3 expression in the lungs of animals with pulmonary hypertension.^43^^,^^44^ Endothelin-1–induced erectile dysfunction depends on Recombinant Staphylococcus aureus Exfoliative toxin A- and Recombinant Staphylococcus aureus Exfoliative toxin B-mediated activation of NLRP3 in mouse corpus cavernosum via Ca^2+^-dependent reactive oxygen species generation.^45^ In vitro study, polymerase chain reaction and Western blotting results showed that after Sil intervention, the expression of eNOS in endothelial cells increased and the expression of inflammatory cytokine IL-18 and pyroptosis-related factors was inhibited. Therefore, our study is the first to demonstrate that Sil improves endothelial dysfunction by blocking the NLRP3/caspase-1 pyroptosis pathway.

Our findings confirmed pyroptosis of penile cavernous cells in HFD-induced ED rats and established how Sil can enhance erectile function by preventing pyroptosis. The study does have some drawbacks, though. First of all, the multistep, progressively proceeded, and carefully controlled process of IL-18 activation is still being further investigated, as is the crucial regulation mechanism of the NLRP3/caspase-1 signal. Second, the findings show that a HFD only causes ED through the traditional pyroptosis pathway; however, it is still unknown whether nontraditional pathways are involved in the pathogenesis of ED.

## Supplementary Material

FIG_8_qfad044

FIG_9_qfad044

FIG_10_qfad044

FIG_11_qfad044

FIG_12_qfad044

Supplement_Figure_Legends_qfad044

## Data Availability

The data generated in the present study are included in the figures and/or tables of this article.
