# Supplementary figures and images for: Exploring the application of sildenafil for high-fat diet-induced erectile dysfunction based on interleukin-18-mediated NLRP3/Caspase-1 signaling pathway
Source: Sex Med. 2023 Aug 25;11(4):qfad044. doi: 10.1093/sexmed/qfad044 (PMC10460117; doi:10.1093/sexmed/qfad044)

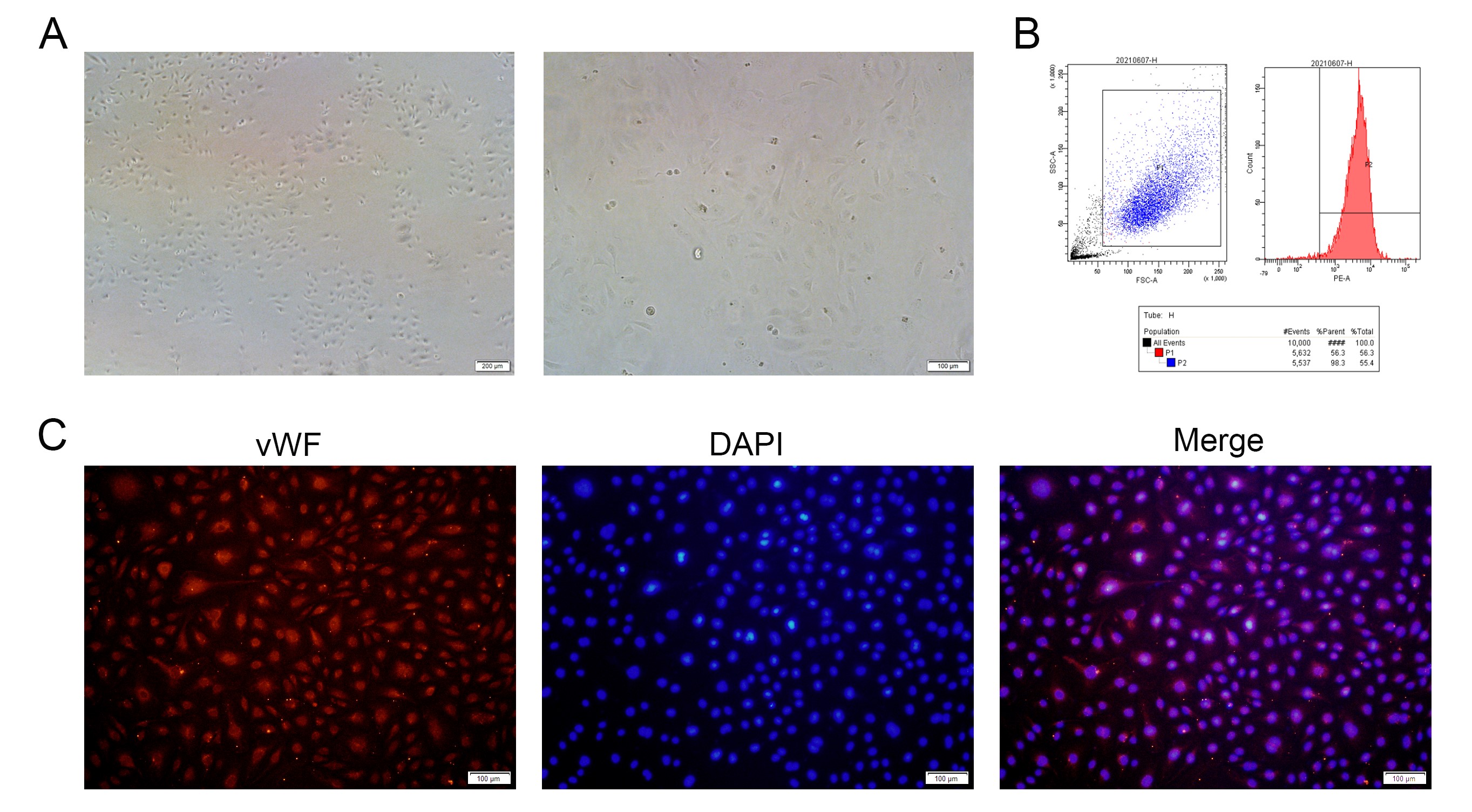

Supplement: FIG_8_qfad044 [file fig_8_qfad044.jpeg]

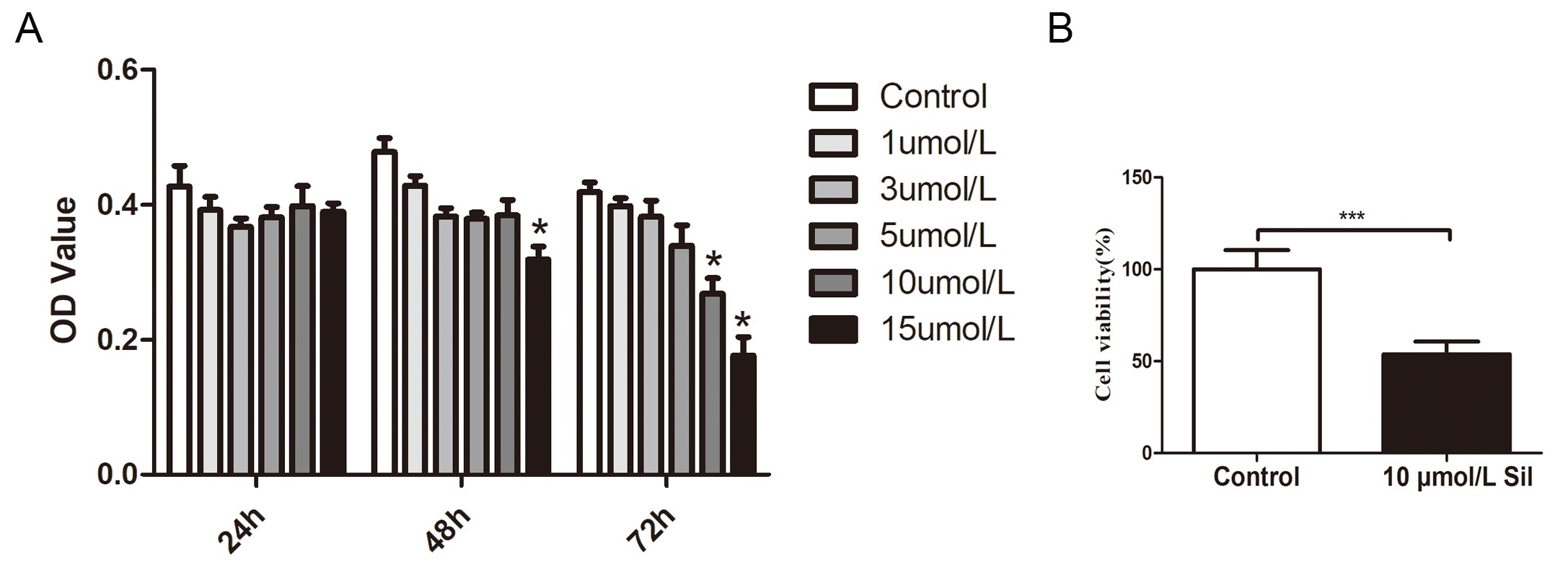

Supplement: FIG_9_qfad044 [file fig_9_qfad044.jpeg]

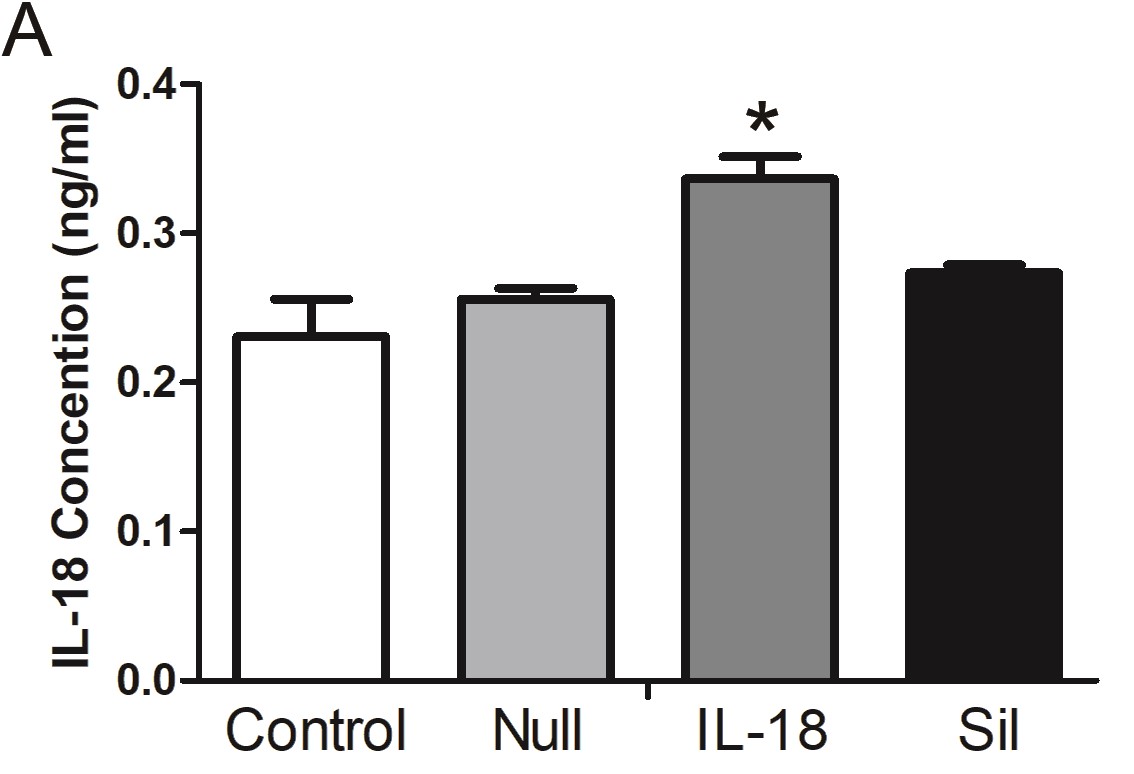

Supplement: FIG_10_qfad044 [file fig_10_qfad044.jpeg]

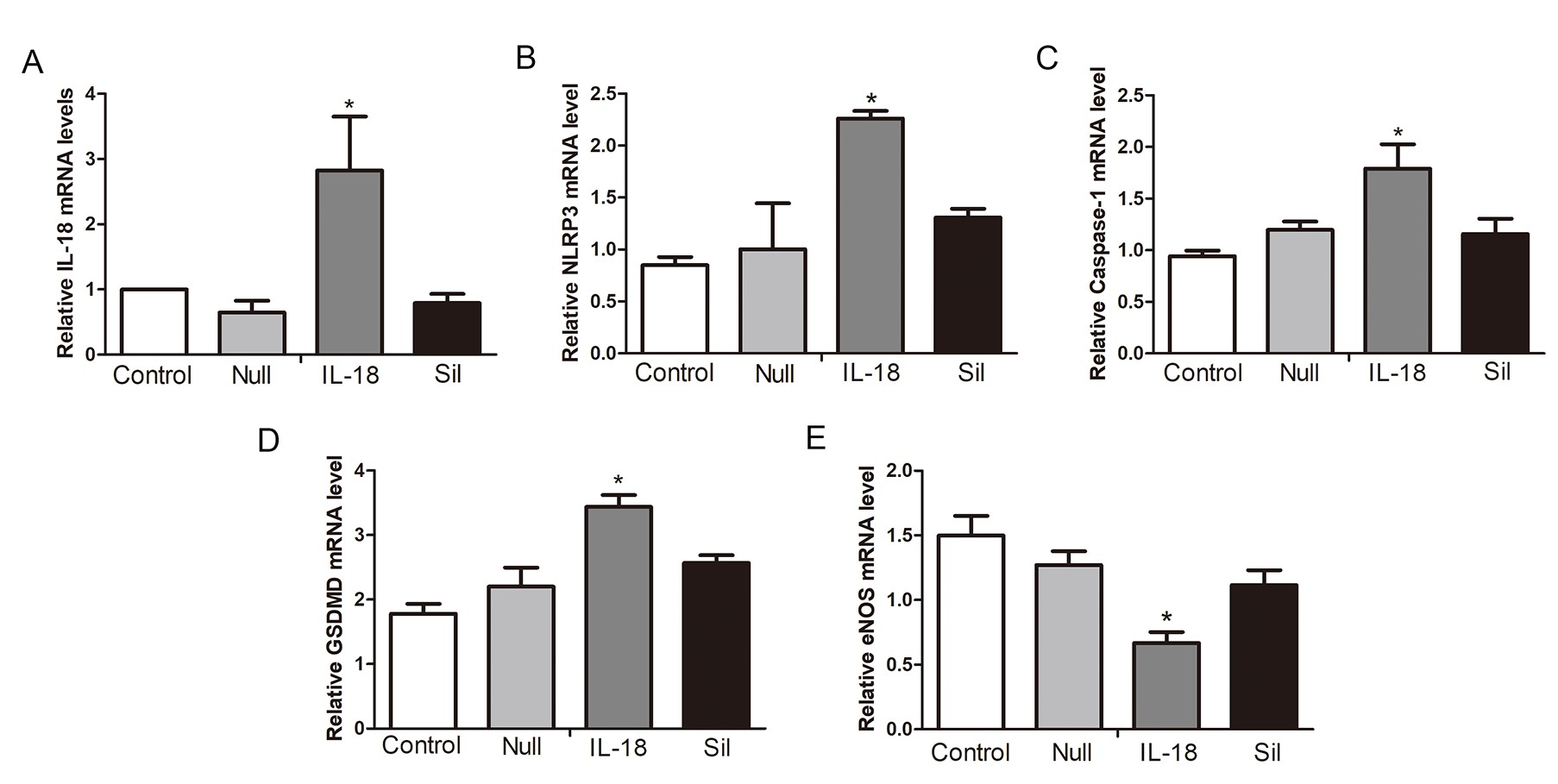

Supplement: FIG_11_qfad044 [file fig_11_qfad044.jpeg]

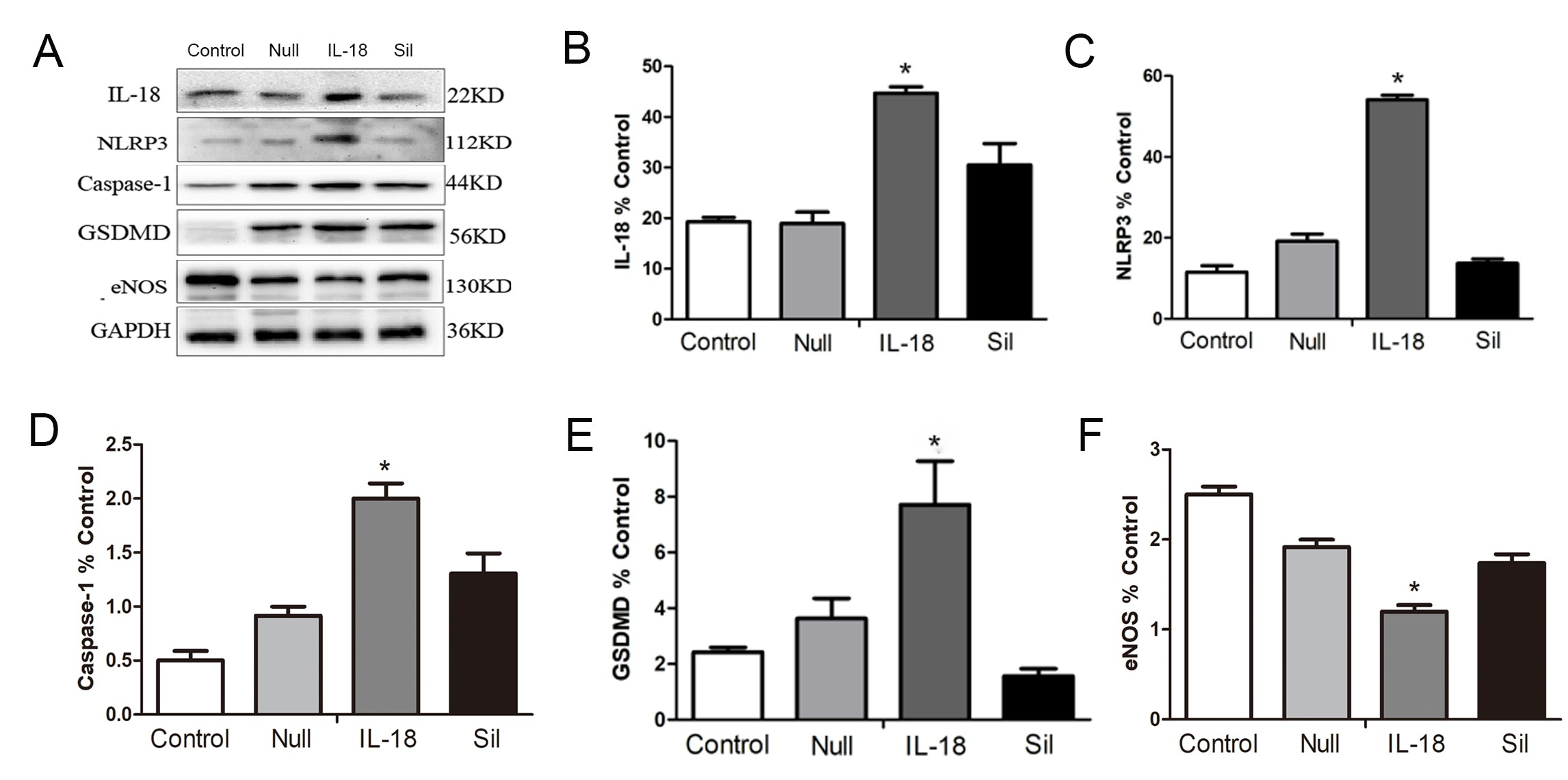

Supplement: FIG_12_qfad044 [file fig_12_qfad044.jpeg]
