# Supplementary material for: Exploring the application of sildenafil for high-fat diet-induced erectile dysfunction based on interleukin-18-mediated NLRP3/Caspase-1 signaling pathway
Source: Sex Med. 2023 Aug 25;11(4):qfad044. doi: 10.1093/sexmed/qfad044 (PMC10460117; doi:10.1093/sexmed/qfad044)
Supplement: Supplement_Figure_Legends_qfad044 [file supplement_figure_legends_qfad044.docx]

**Figure 8 Characterization of cultured HUVECs.** (A) The adherent cultured HUVECs exhibited cobblestone-like morphology. (B) Flow cytometry analysis demonstrated these HUVECs was strongly positive for CD31. (C) Immunofluorescence staining of CD31 (red) and DAPI (blue) in the HUVECs. Scale bar: 100 μm.

**Figure 9 MTT assay for the effect of different times and concentrations of sildenafil on HUVECs.** (A) As the concentration of sildenafil increased over time, the number of cells reduced in comparison to the control group. (B) Cell activity results showed that 10 μmol/L sildenafil was selected for intervention. *Significant difference compared to the control group (*P*<0.05).

**Figure 10 Detection of IL-18 expression between groups of HUVECs by ELISA.** Compared with the control group, the expression of IL-18 in the cell supernatant was significantly increased in the IL-18 group. *Significant difference compared to the control group (*P*<0.05).

**Figure 11 RT-qPCR detection of pyroptosis-related factors and eNOS mRNA levels in each group of HUVECs.** RT-qPCR quantification (A-E) of the mRNA levels IL-18, NLRP3, Caspase-1, GSDMD and eNOS in HUVECs of each group. The IL-18 groups showed increase IL-18, NLRP3, Caspase-1, GSDMD mRNA levels and decrease eNOS mRNA levels in comparison to the control group. *Significant difference compared to the control group (*P*<0.05).

**Figure 12 Western-blotting detection of pyroptosis-related factors and eNOS protein expressions in each group of HUVECs.** HUVECs attenuated pyroptosis through the NLRP3/Caspase-1 pathway in vitro. Representative immunoblot (A) and quantification (B-F) of IL-18, NLRP3, Caspase-1, GSDMD and eNOS in HUVECs of each group. The IL-18 groups showed increase IL-18, NLRP3, Caspase-1, GSDMD protein expressions and decrease eNOS protein expression in comparison to the control group. *Significant difference compared to the control group (*P*<0.05).
